# RNA-Based Multiplexing Assay for Routine Testing of Fusion and Splicing Variants in Cytological Samples of NSCLC Patients

**DOI:** 10.3390/diagnostics11010015

**Published:** 2020-12-23

**Authors:** Cristina Aguado, Ana Giménez-Capitán, Ruth Román, Sonia Rodríguez, Núria Jordana-Ariza, Andrés Aguilar, Carlos Cabrera-Gálvez, Carlos Rivas-Corredor, Pilar Lianes, Santiago Viteri, Irene Moya, Miguel A. Molina-Vila

**Affiliations:** 1 Laboratorio de Oncología, Pangaea Oncology, Hospital Quirón Dexeus, 08028 Barcelona, Spain; caguado@panoncology.com (C.A.); agimenez@panoncology.com (A.G.-C.); rroman@panoncology.com (R.R.); srodriguez@panoncology.com (S.R.); njordana@panoncology.com (N.J.-A.); 2 Instituto Oncológico Dr Rosell, Hospital Quirón Dexeus, 08028 Barcelona, Spain; aaguilar@oncorosell.com (A.A.); sviteri@oncorosell.com (S.V.); 3 Instituto Oncológico Dr. Rosell, Teknon Medical Center, 08022 Barcelona, Spain; ccabrera@oncorosell.com; 4 Medical Oncology Department, Hospital de Mataró, 08304 Barcelona, Spain; crivas@csdm.cat (C.R.-C.); plianes@csdm.cat (P.L.); 5 Instituto Oncológico Dr. Rosell, Hospital General de Catalunya, 08195 Sant Cugat del Vallès, Spain; imoya@oncorosell.com

**Keywords:** cytology, nCounter, NSCLC

## Abstract

The detection of ALK receptor tyrosine kinase (ALK), ROS proto-oncogen1, receptor tyrosine kinase (ROS1), ret proto-oncogen (RET), and MET proto-oncogen exon 14 skipping (*MET*Δ*ex14*) allows for the selection of specific kinase inhibitor treatment in patients with non-small cell lung cancer (NSCLC). Multiplex technologies are recommended in this setting. We used nCounter, a multiplexed technology based on RNA hybridization, to detect ALK, ROS1, RET, and *MET*Δ*ex14* in RNA purified from cytological specimens (*n* = 16) and biopsies (*n* = 132). Twelve of the 16 cytological samples (75.0%) were evaluable by nCounter compared to 120 out of 132 (90.9%) biopsies. The geometrical mean (geomean) of the housekeeping genes of the nCounter panel, but not the total amount of RNA purified, was significantly higher in biopsies vs. cytological samples. Among cytological samples, we detected ALK (*n* = 3), *METΔex14* (*n* = 1) and very high MET expression (*n* = 1) positive cases. The patient with *MET*Δ*ex14* had a partial response to tepotinib, one of the patients with ALK fusions was treated with crizotinib with a complete response. Cell blocks and cytological extensions can be successfully used for the detection of fusions and splicing variants using RNA-based methods such as nCounter.

## 1. Introduction

Although lung cancer continues to be one of the tumors with the highest mortality rate in the world, in recent years, the identification of specific alterations in various genes has allowed the development of targeted therapies, which have improved the clinical outcome of selected groups of patients. Mutations in the EGFR gene and the *MET*Δ*ex14* splicing variant associate with responses to EGFR and MET tyrosine kinase inhibitors, respectively (EGFR- and MET-TKIs) [1,2,3], while fusions involving ALK and ROS1 genes predict a clinical benefit from crizotinib, alectinib, and other ALK tyrosine kinase inhibitors (ALK TKIs) [4,5].

The need to test all markers associated with therapeutic options and the fact that a significant number of patients with non-small cell lung cancer (NSCLC) only have cytological samples available for molecular diagnosis makes urgent the development of multiplexed techniques that can detect clinically relevant markers with a minimum amount of sample. The most widely used multiplexed methodologies for the detection of mutations, amplifications, and fusions in formalin-fixed, paraffin embedded (FFPE) biopsies are DNA and RNA based next generation sequencing (NGS) platforms such as those developed by Illumina, Agilent or Qiagen. However, other methodologies have recently been employed, including mass array and direct hibrydization techniques such as nCounter and the Nanostring platform, which has the advantages of not depending on enzymatic amplification reactions, being an easy technology, and allowing the analysis of low quantity samples. Although several reports have described the use of nCounter for the detection of ALK, ROS1, and RET fusions in FFPE samples [6,7,8], only one of them specifically described results of fusion testing in cytological samples, while *MET* alterations were not assayed [8].

We have previously reported the use of nCounter for simultaneous detection of fusions in ALK, ROS1, and RET [9,10] and, more recently, for *MET* alterations [11]. In this article, we analyze the prospective use of the nCounter technology for the detection of fusions, *MET*Δ*ex14* splicing and MET very high expression in NSCLC cytological samples and we compare the results with FFPE biopsies.

## 2. Materials and Methods

### 2.1. Patient and Cell Line Samples

Samples were obtained from the Quirón Dexeus University Hospital, Hospital General de Cataluña, Teknon Medical Center and Hospital de Mataro with previous informed patient consent. The study had been approved by the ethical committees of each hospital (approval number 51/2018, 1 May 2018) and was conducted according to the Declaration of Helsinki. Formalin-fixed, paraffin embedded (FFPE) 4 μm slides, obtained by standard procedures, were stained with hematoxylin and eosin. A pathologist determined the tumor areas and evaluated the percentage of tumor infiltration, which were subsequently macro- or micro-dissected. For nCounter analysis, RNA was extracted with a high purity FFPET RNA isolation kit (Roche Diagnostics, Mannheim, FRG) according to the manufacturer’s instructions. RNA concentration was estimated using the Qubit 3.0 fluorometer (Invitrogen, Eugene, OR, USA).

### 2.2. nCounter Elements Assay for ALK, ROS1, RET Gene Fusions and MET Alterations

nCounter (NanoString Technologies Inc., Seattle, WA, USA) is a fluorescence-based technique for multiplexed digital transcript profiling without enzyme requirements, amplification, or generation of cDNA. Total RNA (between 5–250 ng) was hybridized with a custom-designed mixture (“codeset”) of biotinylated capture tags and fluorescently labeled reporter probes (Elements Chemistry, Nanostring, Technologies, Seattle, WA, USA). The codeset also contained probes for housekeeping genes, positive and negative controls (Table 1). All processes of hybridization, capture, cleanup, and digital data acquisition were performed with nCounter Prep Station^®^ and Digital Analyzer^®^ (NanoString Technologies) according to the manufacturer’s instructions. Reporter counts were collected with the nSolver analysis software version 3.0. Samples were considered not evaluable if the geometrical mean (geomean) of counts corresponding to the housekeeping genes was lower than 300. Counts from all probes were normalized in two steps and samples were categorized according to previously described threshold values [9].

### 2.3. DNA Purification and NGS

The GeneRead DNA FFPE Kit (Qiagen, Hilden, Germany) was used for DNA extraction from FFPE samples, as described [12]. DNA-based NGS was performed with the GeneRead^®^ QIAact Custom DNA UMI Panel (Qiagen, Hilden, FRG), which can detect mutations in *ALK*, *BRAF*, *CDK4*, *CDK6*, *EGFR*, *ERBB2*, *ERBB4*, *FGFR1*, *IDH1*, *IDH2*, *KIT*, *KRAS*, *MET*, *NRAS*, *PDGFRA*, *PIK3CA*, *RICTOR*, *ROS1*, *STK11*, *TP53*, and copy number variations (CNVs) in *BRAF*, *CDK4*, *CDK6*, *EGFR*, *FGFR1*, *ERBB2/HER2*, *KRAS*, *MET*, *RICTOR*. For the GeneRead panel, up to 40 ng of purified DNA were used as a template. Clonal amplification was performed on 625 pg of pooled libraries and, following bead enrichment, the GeneReader instrument was used for sequencing. Qiagen Clinical Insight Analyze (QCI-A) software was employed to align the read data and call sequence variants, which were imported into the Qiagen Clinical Insight Interpret (QCI-I) web interface for data interpretation and generation of final custom report.

### 2.4. RT-PCR for ALK and MET

RNA was retrotranscribed with the M-MLV retrotranscriptase (ThermoFisher Scientific, Waltham, MA, USA) and random primers. HotStart Taq polymerase (Qiagen) was used for *MET*Δ*ex14* and *EML4-ALK* amplification using a 20 µL reaction (45 cycles) and visualized in agarose gels, as described [9,11]. Positive samples were confirmed by bidirectional Sanger sequencing of RT-PCR products, using the big-dye 3.1 sequencing kit (Applied Biosystems, Foster City, CA, USA).

### 2.5. FISH and IHC

FISH for ALK, ROS1 and MET was performed on 4 μm sections using the FDA-approved Vysis LSI ALK Dual Color Break Apart Probe (Abbott Molecular Inc., Des Plaines, IL, USA), the ZytoLight^®^ SPEC ROS1 Dual Color Break Apart Probe (ZytoVision, Bremerhaven, Germany) and ZytoLight^®^ SPEC *MET/*centromere 7 (*MET*/CEP7) Dual Color Probe (ZytoVision, Bremerhaven, Germany) and, according to manufacturer’s instructions. MET immunostaining was performed with SP44 clone (Roche, Mannheim, Germany) and ALK with the FDA-approved Ventana anti-ALK rabbit monoclonal primary antibody (Clone D5F3, Ventana Medical Systems, Tucson, AZ, USA). In both cases, a BenchMark ULTRA automated tissue staining system was used (Ventana Medical Systems, Valle del Oro, AZ, USA). Membrane staining was graded as described [9,11].

## 3. Results

### 3.1. Sample Characterization

**Figure 1:**
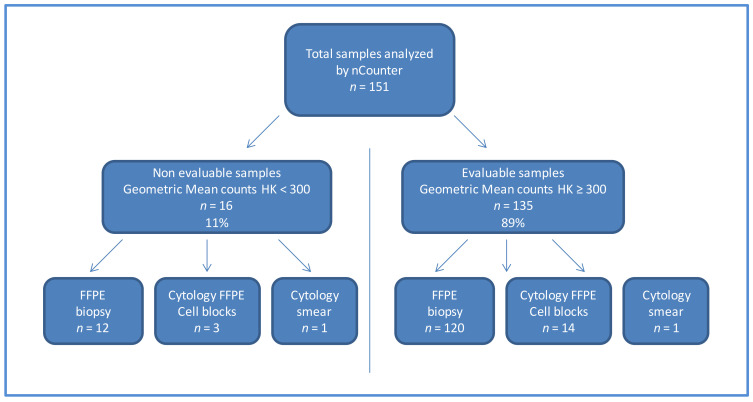
Flow chart of the samples included in the study. Abbreviation: FFPE: formalin-fixed paraffin-embedded HK: housekeeping.

From January 2017 to December 2019, we analyzed 151 samples by nCounter, including FFPE biopsies, cell blocks and cytological smears. Of them, 16/151 (11%) did not meet the quality requirements on nCounter analysis (geomean of housekeeping counts < 300) and were considered non evaluable. Three non-evaluable samples were cell blocks, one was a cytological smear and the remaining 12 were FFPE biospsies (Figure 1).

**Figure 2:**
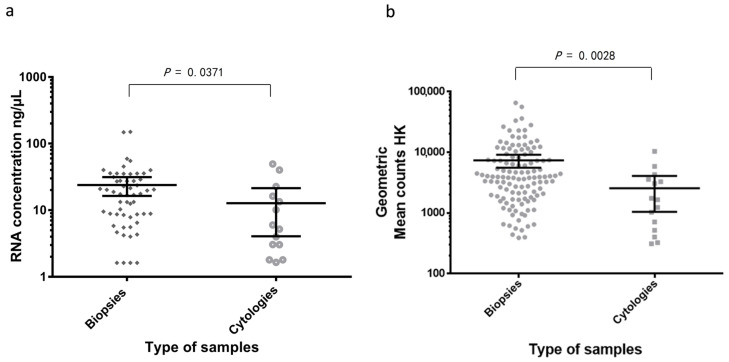
(**a**) Comparison of RNA concentration in ng/µL in biopsies (*n* = 56) vs. cytologies (*n* = 14). (**b**) Comparison of the geomean of housekeeping (HK) mRNA levels in biopsies (*n* = 120) and cytologies (*n* = 15).

Among the valid samples (*n* = 135), 120 corresponded to FFPE biopsies, 14 were cell blocks, and one a cytological smear. All 15 evaluable cytological samples had more than 40% of tumor cells and presented adenocarcinoma histology. After purification, RNA concentration ranged between 1.65–49.3 ng/µL, as quantified by Qubit (Table 2 and Table 3). The FFPE biopsies showed a tumor infiltration between 5% and 85%; adenocarcinomas represented 80% of cases and RNA concentration was measured in 56/135 specimens, ranged from 1.65 to148 ng/µL, as measured with Qubit. The RNA was significantly different between cytological samples (*n* = 14) and biopsies (*n* = 56) (*p* = 0.037 in a Mann–Whitney test; Figure 2a). Also, upon nCounter analysis, the geometric mean of the endogenous gene counts was significantly higher in the biopsies (*n* = 120) vs. cytological samples (*n* = 15) (*p* = 0.0028 in a Mann–Whitney test; Figure 2b).

### 3.2. nCounter Results of Cytological Samples and Biopsies

**Figure 3:**
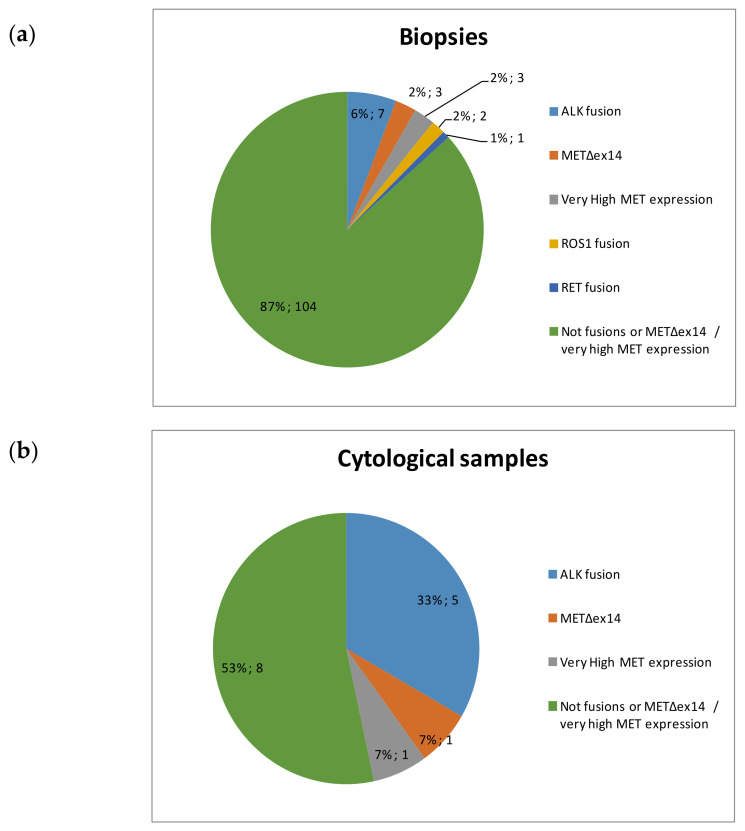
Distribution of alterations detected by nCounter in (**a**) biopsies (**b**) cytological samples.

The only evaluable cytological extension did not present fusions or *MET*Δ*ex14* transcripts. Among the 14 FFPE cell blocks, nCounter detected alterations in 7 (50%), including ALK fusions (*n* = 5), *MET*Δ*ex14* transcripts (*n* = 1) and very high levels of *MET* mRNA expression (*n* = 1). In contrast, among the 120 valid biopsy samples, 16 (13%) harbored ALK (*n* = 7), ROS1 (*n* = 2), or RET fusions (*n* = 1), and *MET*Δ*ex14* (*n* = 3) or very high MET expression (*n* = 3) by nCounter (Figure 3).

**Figure 4:**
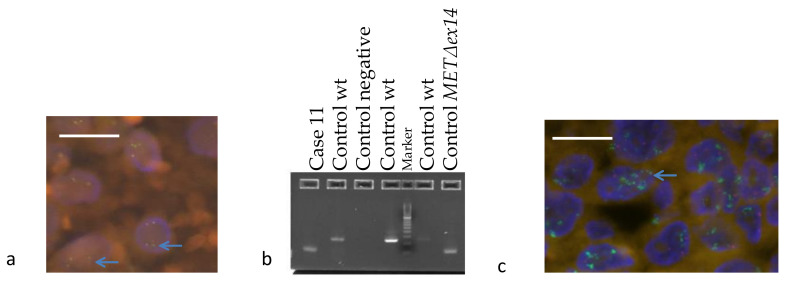
(**a**) Fluorescence in situ hybridization analysis for *ALK* in sample 10, blue arrows indicate translocated signals; (**b**) Visualization by agarose gel of the PCR band corresponding to the *MET*Δ*ex14* splicing variant in sample 11. The upper band corresponds to the amplification of the wt region. (**c**) Fluorescence in situ hybridization analysis for *MET* in sample 7, blue arrows indicate amplified cells. The scale bars correspond to 20 µm.

Four ALK cytological samples were positive by 5′/3′ imbalance and specific fusion probes; three corresponded to variant 1 and the remaining sample to variant 3. The other ALK cytological sample was positive only by 5′/3′ imblance (Table 4). Three of the ALK cases could be confirmed by IHC and another by FISH, while the specimen with the *MET*Δ*ex14* was confirmed by RT-PCR and NGS (Figure 4). The sample with very high MET expression was submitted to IHC, FISH and NGS. The results were *MET* amplification by both FISH and NGS (ratio MET/CEP7 > 5 and copy number = 25.12, respectively) and high protein expression (histoscore = 240), as expected (Figure 4 and Table 4).

Finally, 11 out of 15 cytological samples yielded valid results by NGS. Amplifications in EGFR (*n* = 2), ERBB2 (*n* = 2) and CDK4 (*n* = 1) and mutation in ERBB2 (*n* = 1) were detected, which were mutually exclusive with fusions and MET skipping variant. No mutations in driver genes such as KRAS or EGFR were found.

### 3.3. Response to TKIs

Clinical data were available from four patients in which ALK or MET alterations were detected in cytological samples. One patient with *MET*Δ*ex14* was treated with MET tepotinib and had a partial response. Regarding *ALK*-positive patients, three were treated with TKIs and derived clinical benefit, two complete responses (to crizotinib and alectinib) and one partial response (to alectinib).

## 4. Discussion

The use of multiplexed panels for the detection of mutations and fusions in the lung cancer clinical environment has been exponentially increasing over the last few years [10,13]. Previous studies have shown that cytological smears and cell blocks are suitable for performing DNA and RNA massive sequencing [14,15,16]. In the 2018, the Molecular Testing Guideline for the selection of Lung Cancer patients for treatment with targeted tyrosine kinase inhibitors included a recommendation for the use of cell blocks and other cytologic preparations as suitable specimens for biomarker molecular testing. The Guideline strongly recommends ALK and ROS1 testing in all NSCLC samples, and presents expert consensus group opinions suggesting the use of multiplex testing panels for testing RET fusions and MET alterations in the context of clinical trial [17].

The nCounter platform is increasingly being used for fusion detection in NSCLC and other types of tumors [18,19,20,21]. However, there is only one report in the literature describing the performance of nCounter in cytological samples, and MET alterations were not tested [8]. In consequence, we decided to investigate if cytological samples can be used for multiplex detection of fusions and splicing variants in NSCLC. We found that, although both, the mRNA levels of housekeeping genes and the mRNA concentrations are lower in cytological samples compared to biopsies, they are generally sufficient for multiplex analysis of fusions and MET alterations by nCounter. In our study, 10% of FFPE biopsies were non evaluable by nCounter vs. 17.6% for cell blocks. Regarding cytological smears, only two were tested and one of them (50%) was not assessable. The higher percentage of non-evaluable cytology samples is probably a consequence of the lower amounts of tumor cells present compared to biopsies. These results endorse the incorporation of RNA-based testing of fusions in cytological samples into the routine of clinically-oriented molecular laboratories. This testing could be particularly useful in patients at progression, where sampling is frequently performed by FNA or related techniques.

In our cohort of 104 FFPE biopsies we found 6% ALK positive samples, 4% of specimens with MET alterations and 3% of RET and ROS1 fusions (Figure 3). These percentages are in line with those described in the literature when using nCounter or other methodologies in advanced NSCLC samples [22,23]. In contrast, five out of 15 (33%) cytological specimens harbored ALK fusion transcripts, and 2/15 (13%) MET alterations. These frequencies are significantly higher than those described in NSCLC populations and we did not detect any single case of EGFR or KRAS mutations in our series, strongly suggesting a biased population. Regarding the reason of this bias, the percentage of smokers and former smokers in our cytologies and biopsies was similar. Also, mutations in EGFR have been described to be associated with pleural retraction and ALK fusions with pleural effusion, but almost all our cytological samples corresponded to FNAs [24]. Finally, the incidence of lymph node metastasis has been found to be significantly higher in ALK-positive (45.1%) compared with wild-type, KRAS, and EGFR-mut NSCLC [25], and a significant percentage of our FNAs corresponded to lymph node metastasis (33%; Table 3), a fact that could partly explain the observed bias. Among the 120 FFPE biopsies of our series, only five (4.2%) were taken from lymph nodes. Remarkably, two of them were fusion-positive for ALK (*n* = 1) and RET (*n* = 1).

## 5. Conclusions

Cell blocks and cytological extensions can be successfully used for the detection of fusions and splicing variants using a multiplexed RNA-based hybridization technology such as nCounter.

## Figures and Tables

**Table 1:** Description of the nCounter codeset used in the study.

Probes	Number
Gene Fusions Probes (*ALK*, *ROS1*, *RET*, *NTRK1*)	29 pairs
Imbalance Probes (*ALK*, *ROS1*, *RET*, *NTRK1*)	32 pairs
Housekeeping Genes Probes (*ACTB*, *GAPDH*, *PSMC4*, *MRPL19*)	3 pairs
MET Gene Probes (*MET*Δ*ex14*, MET wild type)	2 pairs

**Table 2:** Characteristics of the valid samples included in the study.

Characteristics	Cytological Samples (*n* = 15)	Biopsies (*n* = 120)
Histological type		
Adenocarcinoma	15	96
Squamous cell carcinoma	0	10
Other	0	13
Unknown	0	1
UICC stage		
I-IIIA	0	13
IIIB-IV	12	84
Unknown	3	23
Collection time		
Basal	10	73
Progression	3	11
Unknown	2	36

**Table 3:** Characteristics of the valid cytological samples included in the study.

Case Number	Sample Type	Procedure	Organ	% of Tumor Cell	Histology	RNA ng/µL	GeoMean Housekeeping
1	Cytologic smear	nd	nd	60	ADK	5.2	1027
2	Cell Block	FNA	Lymph node	80	ADK	40	3043
3	Cell Block	FNA	Suprarrenal	85	ADK	13.4	3250
4	Cell Block	FNA	Lung	75	ADK	1.65	399
5	Cell Block	nd	nd	40	ADK	22.6	4282
6	Cell Block	FNA	nd	90	ADK	49.3	10,358
7	Cell Block	FNA	Lung	50	ADK	1.8	311
8	Cell Block	FNA	ND	70	ADK	16.2	3575
9	Cell Block	nd	Lung	50	ADK	1.8	323
10	Cell Block	FNA	Lymph node	80	ADK	3.05	1650
11	Cell Block	FNA	Lung	50	ADK	nd	5838
12	Cell Block	FNA	Lymph node	40	ADK	6	343
13	Cell Block	FNA	Lymph node	40	ADK	10.1	1232.98
14	Cell Block	FNA	Lung	60	ADK	3.99	516.98
15	Cell Block	FNA	Lymph node	90	ADK	3.04	1738.58

Abbreviation: ADK, adenocarcinome; FNA, fine-needle aspiration; nd, non detected.

**Table 4:** Genotying results of the cytological samples.

Case Number	nCounter Result	FISH Genes/Result	IHC Genes/Result	RT-PCR Genes/Result	Other Results NGS Genes/Result
1	Wt	na	na	na	*ERBB2*/Amplification copies = 11
2	Wt	na	na	na	*ERBB2*/Amplification copies = 9
3	Wt	*ALK*/No fusión; *ROS1*/No fusión; *MET*/No amplification	ALK/Neg; MET/Neg	*MET*Δ*ex14*/nd	nd
4	*ALK* fusión (5′/3′, v1)	*ALK*/n v	ALK/95% 3+	*MET*Δ*ex14*/nd; *ALK* v1, v2, v3/detected v1	nd
5	Wt	na	ALK/Neg; MET/Neg	*MET*Δ*ex14*/nd	nd
6	Wt	na	na	*MET*Δ*ex14*/nd	*EGFR*/Amplification copies = 11
7	Very high MET expression	*MET*/Amplification r > 5	MET/80% 3+	*MET*Δ*ex14*/nd	*MET* Amplification copies = 25.1
8	Wt	na	na	na	*EGFR*/Amplification copies = 7
9	*ALK* fusión (5′/3′, v1)	na	na	na	na
10	*ALK* fusión (5′/3′, v3)	*ALK/*Fusión 58%	na	*MET*Δ*ex14/*nd	nd
11	*METΔex14*	na	na	*MET*Δ*ex14*/detected	*MET*Δex14
12	Wt	na	na	*MET*Δ*ex14/*nd	*CDK4*/Amplification; *TP53*/p.R248W
13	Wt	na	na	na	*ERBB2* p.S335P; *TP53* p.V157F
14	*ALK* fusión (5′/3′, v1)	na	ALK/Pos	na	na
15	*ALK* fusión (5′/3′, nd)	na	ALK/Pos	na	na

Abbreviation: FFPE: formalin-fixed paraffin-embedded, HK: housekeeping, na: non analyzed, nd: non detected, Neg: negative, nv: non evaluable.

## Data Availability

The data presented in this study are available within the article.
